# Texting in a crisis—using SMS for information and emotional support during COVID-19: A mixed methods research study

**DOI:** 10.3389/fsoc.2022.1053970

**Published:** 2022-11-30

**Authors:** Mengdi Wang, Changzheng Wang, Xiaobing Peng

**Affiliations:** ^1^School of Public Policy and Administration, Chongqing University, Chongqing, China; ^2^School of Public Policy and Administration, Chongqing Technology and Business University, Chongqing, China

**Keywords:** short message service (SMS), COVID-19, emotional support, grounded theory, traditional media

## Abstract

In the era of new media, short message service (SMS) is no longer seen as advantageous and it is no longer used very much by the Chinese public. However, as a traditional media, local governments managing public health crises used SMS as a way of meeting the public's need for emotional support during the COVID-19 pandemic. Our study examined 108 SMS texts pushed to phones in Chongqing between January and December 2020, and carried out in-depth interviews with ten interviewees. This mixed research method of descriptive and grounded theory analysis was designed to investigate how SMS was used to communicate prevention guidelines and give emotional support during COVID-19. The results show that Chongqing Municipal Health and Health Commission gained the public's attention with SMS messages consisting of neutral, objective advice, and guidance to reduce people's anxiety and panic. However, with the stabilization of COVID-19, SMS has once again been discarded by users, including the public health sector. The study found that the emotional support offered by SMS was limited to the elderly, a subset of the population considered to be weak users of the internet. SMS has been replaced by other technologies, but along with other media, such as official media and social media, it has shaped the media communication environment and served as an emotional support channel for the public. Undoubtedly,the use of SMS during COVID-19 presents a research opportunity for exploring its capacity for prevention, control and emotional support.

## Introduction

Short message service (SMS) is the traditional communication medium of information push technology in the mobile network. It is a product of information transmission during the period of mobile communication development and the developing Internet information network. With the advent of Internet information and the new media era, SMS seem to have disappeared from public life in China. According to the statistics from The Ministry of Industry and Information Technology of China (Zhongming et al., 2017), the phone SMS volume in China was 58,110.7867 million in October 2008, with an increase of 20.1% over the same period of 2007. In the following years, the growth of SMS traffic declined, falling to 6.4% at the end of 2011 and showing negative (−0.6%) growth at the end of 2013. The decline in SMS use in just a few years was due to upgrades in Internet and media technology and the upward trend of China's Internet users. The main reason is that from 2008 to 2013, China realized the transition of the network era from 2G and 3G to 4G. On December 4, 2013, the 4G license was distributed to the three major mobile operators (Mobile, Unicom, and Telecom) and the 4G network business began operations. Meanwhile, in 2011, a Chinese version of WhatsApp, known as WeChat, went online. WeChat is a social media service that allows users to communicate *via* audio, video, and text while relying on web traffic to cut costs. Driven by Internet information technology and new media, SMS disappeared in people's daily life in a short time. According to the *Statistics of China Internet Development Report (2019)* (CNNIC, 2020), the number of Internet users in China reached 800 million by the end of 2018, and the percentage of Internet users accessing the Internet through mobile phones was as high as 98.6%. This phenomenon of increasing network users is happening all over the world where the number of people using the Internet far exceeds the number using SMS (CTIA Wireless Association, 2009).

Whether used as a medium for public emotional support or information exchange, the public's perception of the advantages of SMS has declined. As an intermediary communication medium, SMS can promote “interconnected existence” or “communication preparation” (Licoppe and Smoreda, 2005; Nardi, 2005). People can meet multiple times simultaneously and across time, which facilitates a continuous understanding of other people and relationships, indicates users' availability, and maintains the social climate (e.g., continuous common ground or connection area). These uses in turn influence the way other media are chosen and interpreted (Wei and Lo, 2006). Because of the limited text capacity of SMS, the sender and the receiver need to know how to use text and understand the limited text content, which is not conducive to social contact. Studies have found that people use Internet instant messaging to conduct daily conversations with many friends, a practice that is more social than Face to Face or telephone conversations and can also extend users' social network (Oksman and Turtiainen, 2004; Hyo et al., 2007).

However, SMS have been used to address users' health issues and provide emotional support. Examples of this include weight loss, smoking cessation, community sexual health programs, and rural environment projects (Ryan and Hayden, 2012; Sneha et al., 2014; Cassandra et al., 2016; Chalela et al., 2020). SMS are regarded as a low-cost communication medium for patients and consumers and patients and providers. Meanwhile, SMS ensures anonymity and emotional interaction between participants and provides additional assistance when necessary (Fjeldsoe et al., 2010). However, the effectiveness of SMS is also controversial. Some studies have found that SMS can be effective in managing weight loss (Bauer et al., 2010; Fukuoka et al., 2010), but other studies found no increase in motivation or willingness to exercise or significant effect on weight loss among people who receive exercise text messages (Newton et al., 2009). Similarly, in smoking cessation programs, SMS may have been effective because the participants were mostly light or moderate smokers who did not need a great deal of support to quit (Chalela et al., 2020). The Sexual Health and Youth (SHY) project using SMS had little effect (Cassandra et al., 2016). These studies give us avenues to explore how SMS can be a more useful communication tool.

In China, SMS use is declining. Their simplicity, low cost, and ability to act as a prompt for action (Rice and Katz, 2003) had advantages in the early days of the Internet but the maximum text capacity of 160 characters has downgraded this advantage. SMS has been replaced by faster and more diverse and more capacious forms of the Internet. In addition, the functionality of SMS has shifted so that SMS is no longer popular with the public. The emergence of new media has replaced the function of SMS, with the result that SMS now have new functions and users. Users are no longer the public but enterprises and advertisers who conduct product promotions and user registration, send out push messages and provide verification code services. Even more serious are illegal groups that engage in illegal activities such as sending harassing text messages or viral text messages in group batches. These spam messages have led to a loathing of SMS by citizens and altered their attitude toward SMS. According to a report by the China 360 Company, a company engaged in mobile phone and computer network security (360ISC, 2020), the company intercepted 71.2 billion spam messages for 220 million users, 195 million a day in 2012, through its security software. The spam messages contained information on goods discounts and promotions (43%), while the rest of the messages consisted of fraud and illegal information. By 2017, 360ISC had intercepted about 9.85 billion spam messages, or 26.986000 a day on average. Although the overall trend shows a rapid decline, it was enough to indicate that SMS's original function had been replaced and its media role no longer met users' needs. There are similar situations in other countries where SMS messages on goods, services, and promotional ideas are sent to individuals, groups, or customers. A large number of SMS (one-way communication) on promotions and coupons are sent to customers. However, it is unclear if the sender received permission to send the SMS and whether the illegal content provokes users (Noprisson et al., 2016).

Although the SMS situation in China is unfortunate, short message pushing has been officially recognized by the government and continues to be used for official information propaganda, notice release, and policy delivery. SMS is a form of media-oriented government governance for the government's management of public events, policy requirements, and interaction with the media, especially during public health crises such as COVID-19. This has led to an interesting phenomenon—SMS lost their advantage and became unpopular with the public but are still used by the government for pandemic management. Therefore, it is necessary to revisit the role of SMS in governance and their relationship to the psychological and emotional support needs of the public. Therefore, in this study, we focused on the following research premise: Compared with other media, SMS have the advantage of extensive coverage even though the information they deliver is extremely limited and relatively simple. They have become unpopular and abandoned by the public in the new media era. Why then are SMS still recognized and used for governance? Thus, it is necessary to re-examine and discuss SMS and the public emotional support they offer for the prevention and control of COVID-19.

For this paper, we researched the role of SMS during the height of the Covid period (January–December 2020) in Chongqing, China. During this period, the government pushed pandemic-related SMS data analysis and interview surveys to users' mobile phones. We used descriptive analysis and grounded theory research methods to study the entire process of pandemic prevention and control by the government to discover how SMS, now a traditional rather than innovative media form, provided emotional support to the public during the crisis and helped the public to recover from the traumatic event.

## Materials and methods

### Research methods

This study used a mixed research approach. A mixed research approach enables the collection, analysis, and mixing of quantitative and qualitative data in a study or a series of studies to better understand the research problem. This mixed research approach is the third methodological paradigm after quantitative and qualitative research (Johnson et al., 2007). We adopted a two-stage study: descriptive analysis, semi-structured interviews, and a hybrid study based on grounded theory.

Descriptive analysis is mainly used to describe relevant characteristics or trends by grouping them by different regions, different times or different types, using data (including laboratory test results) obtained from routine testing records or through specialized surveys (Heymann et al., 2014). By collecting the data of Chongqing's SMS use during the COVID-19 period, we conducted a descriptive analysis for both historical review and structural profile (Lawless and Heymann, 2010). The characteristics and process of Chongqing's SMS use were reviewed and described using Excel in the time dimension, and then the process was combined with the specific content of the pushed messages and the relevant pandemic policies of Chongqing city. This process or stage was further analyzed in depth. The problem of *what* is mainly solved.

Immediately following the *why* question, we collected data through semi-structured interviews and adopted a grounded theory approach to interpretation. The grounded theory approach is interpretivist (Cassiani et al., 1996), focusing on the foundations of theory generation in a systematic process of data collection and analysis (Noble and Mitchell, 2016). Zigzag theory, on the other hand, is inductive in nature and allows for subjective interpretation during data analysis, so that researchers can go beyond the collected data and form an interpretation of the context of the data source (Tripathi et al., 2022). To obtain data for the grounded theory analysis, we conducted semi-structured interviews for different ages, occupations and types of interviewees in Chongqing, noting their thoughts, and coding and analyzing them through NVivo 12 software.

### Sample selection and data collection

#### SMS data collection

**Figure 1:**
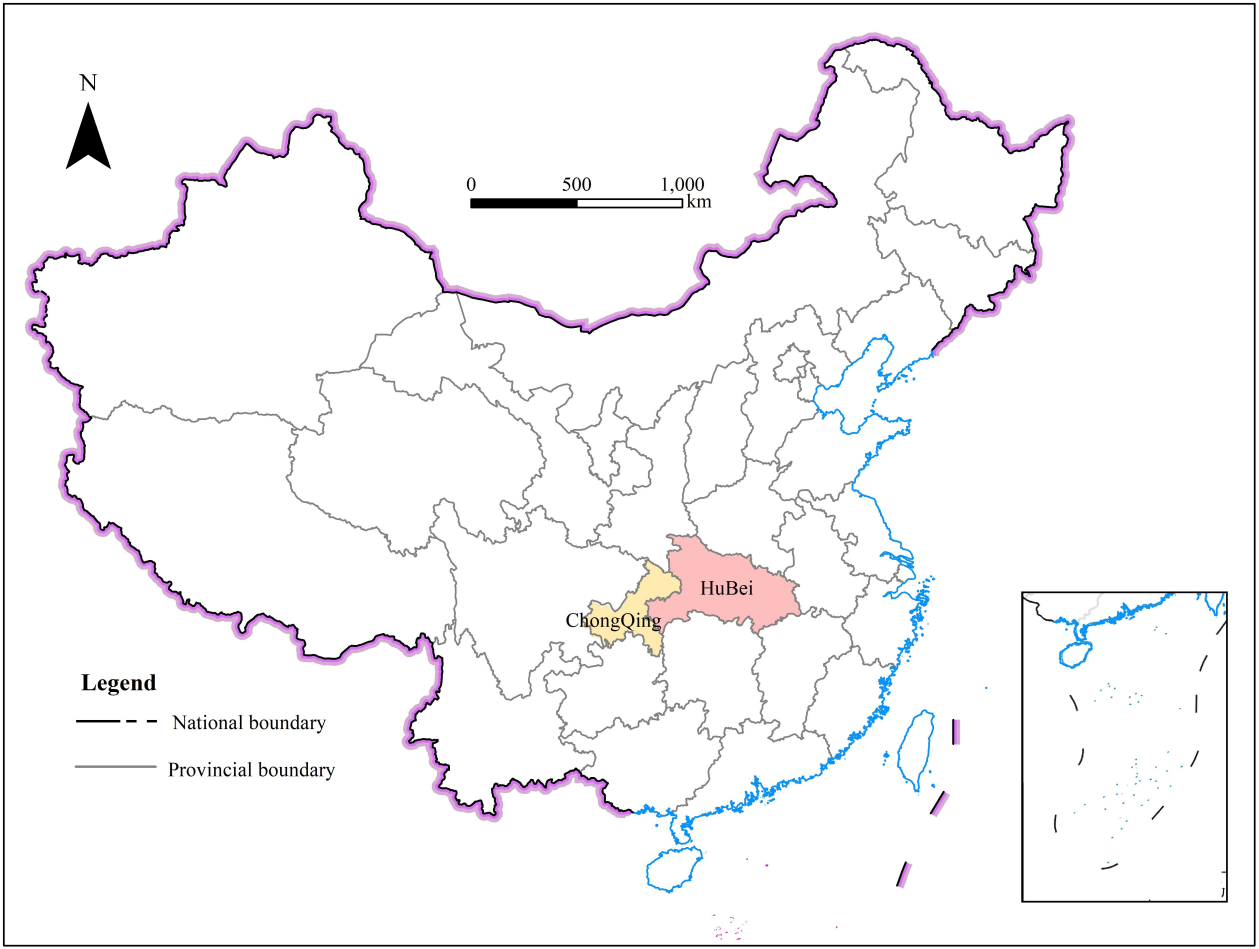
Locations of Chongqing and Hubei.

Chongqing was chosen as the study area for the following reason. Firstly, its geographical location and population density. Chongqing is adjacent to Hubei province, where the outbreak started, and 53 towns and counties in nine districts and counties in Chongqing border with Hubei province (Figure 1). Chongqing has close economic and demographic ties with Hubei province. In addition, Chongqing has the highest population density in China, with an area of 23 km^2^ and a resident population of 32.12 million as of 2021. The resident population growth rate in 2021 was 0.1%, faster than the national average of 0.07%, and had been growing for 17 consecutive years. At the beginning of the COVID-19 pandemic, Chongqing was affected by geographical location and population factors, and cases spread rapidly. It became one of the top ten hardest hit areas in China.

Secondly, Chongqing is one of the four municipalities directly under the central government of China, and has the fourth largest GDP in China and an important economic status. With a total import and export trade of 651.4 billion RMB in 2020, Chongqing is in the top ten in China and has close ties with foreign regions, so it was significant for China's economic and social stability that Chongqing could effectively prevent and control the pandemic.

**Figure 2:**
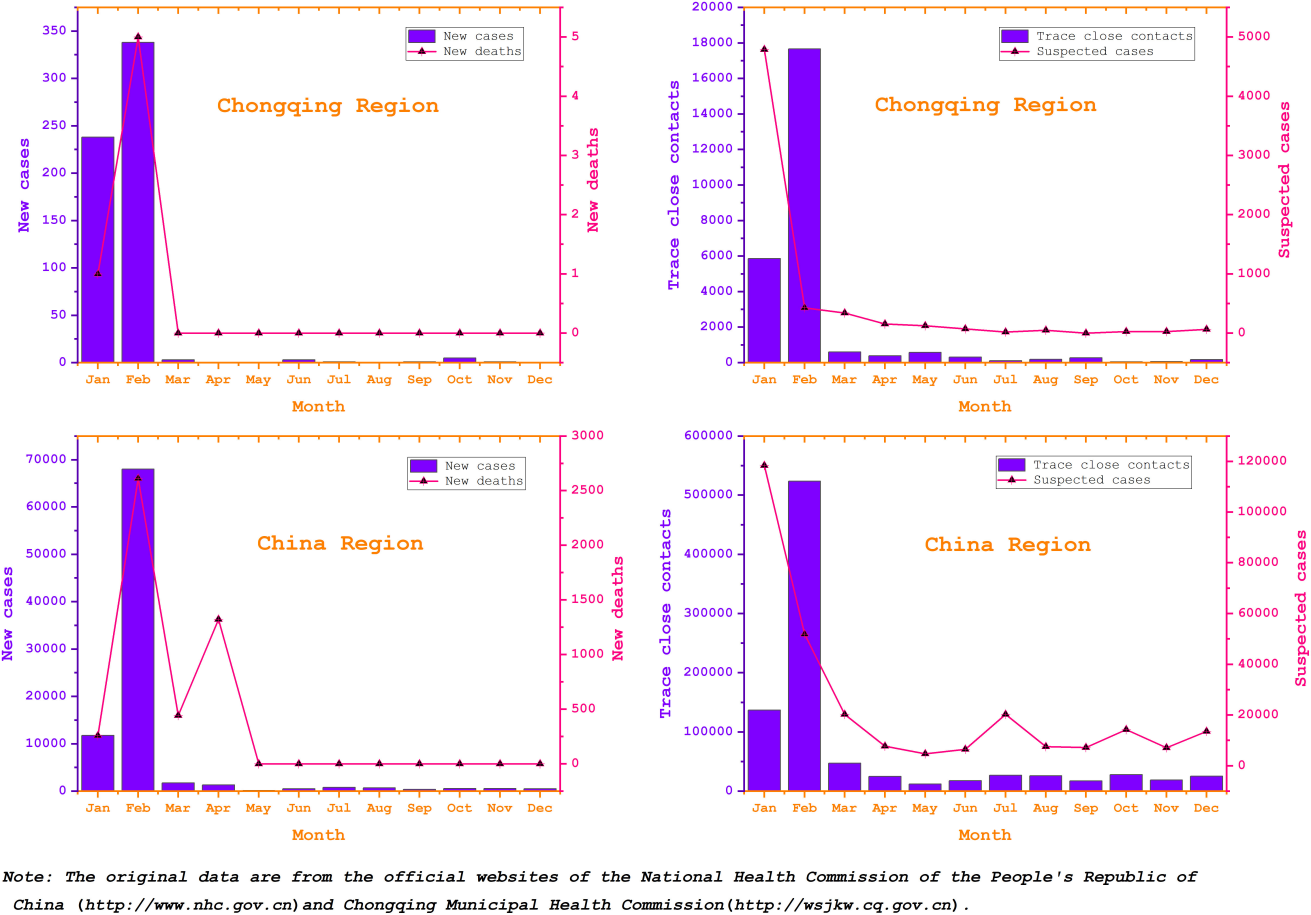
Number of monthly new COVID-19 cases, deaths, trace close contacts and suspected cases in China and Chongqing city.

Thirdly, the researchers and their institutions are based in Chongqing city, making field surveys easier during the pandemic. For these reasons, the study of the relationship between SMS and pandemic prevention and control in Chongqing is representative and operable. Number of monthly new COVID-19 cases, deaths, trace close contacts and suspected cases in China and Chongqing city is shown in Figure 2.

We studied SMS messages sent by the government about pandemic prevention and control in Chongqing between January and December 2020. This was a critical period for the prevention and control of the pandemic in China. It spread rapidly at the beginning of the outbreak, and the strictest and most thorough measures were taken to prevent and control the outbreak. The National Health and Wellness Commission and governments at all levels organized the issuing of guidelines for the prevention and control, actively easing public panic, and sharing prevention and control information. After the effective prevention and control of the pandemic in China, many places lowered the level of public health emergencies from Level 1 to Level 2, comprehensively promoted the resumption of work and school, restored normal economic and social order, and adopted the Yu Kang Code for public pandemic follow-up and news announcements.

In view of the specific changes in prevention and control and the content of SMS messages, we divided our study into two phases: the first phase being containment (January–April 2020), and the second phase normalization of pandemic prevention and control (May–December 2020). During this period, there were SMS messages from both national and local governments, and we focused on the date, content, frequency, and origin to examine the role of SMS messages.

#### Interview data collection

This study addressed the core question of how public sentiment needs could be met during a pandemic and whether SMS messages can support public sentiment needs. We conducted semi-structured interviews based on grounded theory (Corbin and Strauss, 1990; Hsieh and Shannon, 2005; Chung and Seomun, 2021) around the theme of SMS governance of public health crises. These were online in-depth interviews. A small group of representative citizens and expert scholars were selected from the researcher's own social network as interviewees. At the same time, considering the trust base of community workers and government personnel among the public, online one-on-one interviews were conducted *via* WeChat or telephone for urban and rural communities in Chongqing, as well as for the Chongqing Health Commission, selected to participate in the prevention and treatment of the COVID-19 pandemic.

We had a total of ten interviewees. Through the analysis of grounded theory after-interview data collection and according to the principle of information saturation (Martin-Crespo and Salamanca, 2007; Noble and Mitchell, 2016), no more interviewees were added when information saturation was reached. The specific characteristics of the interviewees are shown in Table 1.

**Table 1:** Descriptive statistical analysis of interviewees.

**Interviewee code**	**Sex**	**Age**	**Type**	**Occupation**
1	Female	61	Urban elderly	/
2	Female	62	Rural elderly	/
3	Male	28	Young	Construction worker
4	Female	29	Young	Enterprise executive
5	Male	45	Middle-aged	Construction worker
6	Female	43	Middle-aged	Housewife
7	Male	32	Urban community	Leader
8	Female	41	Rural community	Leader
9	Male	43	Chongqing Health Commission	Government personnel
10	Male	48	University	Professor

Although the interview process began with an interview outline (Table 2), the questions asked were open-ended, and the interviewees' responses were not limited to the interview outline, allowing for more relevant and useful information. We maintained a neutral attitude during the process, recording the interviews in a timely manner without value guidance, and trying to capture new clues. We also informed the interviewees of the purpose of the information collection and of their anonymity. We obtained permission from the interviewees.

**Table 2:** Interview outline.

**Item**	**Interview question**	**Type**
A	What were your sources of information during the pre- and post- COVID-19 outbreak?	Description
B	Did you receive any government messages in the run-up to the COVID-19 outbreak? Were you aware of its content and what did you think of it?	Description and evaluation
C	How did you respond to the government's call to take control of the COVID-19 pandemic in the run-up to the outbreak?	Description
D	Did you get any misinformation? Through what channels did you get it? How do you feel when you heard misinformation? How did you handle and identify the misinformation?	Description and evaluation
E	Did your perception of the COVID-19 pandemic change during the pre- and post- COVID-19 pandemic period? How did it change?	Description
F	What is your assessment of the short messages throughout the prevention and control of the outbreak?	Evaluation
G	In your opinion, how do you rate the way the government pushed out SMS messages during the prevention and control of the COVID-19 outbreak compared to other media?	Evaluation

## Research data analysis

### SMS descriptive analysis

Between January 21, 2020, and December 31, 2020, the Chongqing government sent a total of 108 identical short messages to Chongqing residents (including the interviewees) in response to COVID-19. After collating the data on the time, departments, and content of the short messages sent, we found that within a 12-month cycle, January, February, March, and April were the months with the highest number of sent short messages −11,33,27,12—respectively, followed by a general decreasing trend. Concerning the short messages push departments of the Chongqing Municipal Health Commission, a total of 78 short messages were sent by 72% of the departments. The remaining push departments included the Office of State Security, the Office of Popular Law, the Tourism Bureau, the Civil Affairs Bureau, the Transport Bureau, and 14 other departments. The content of the SMS focused on personal protection (e.g., daily mask protection), policy requirements (e.g., standards for enterprises to resume work), holiday tips, rumor clarification, etc.

**Figure 3:**
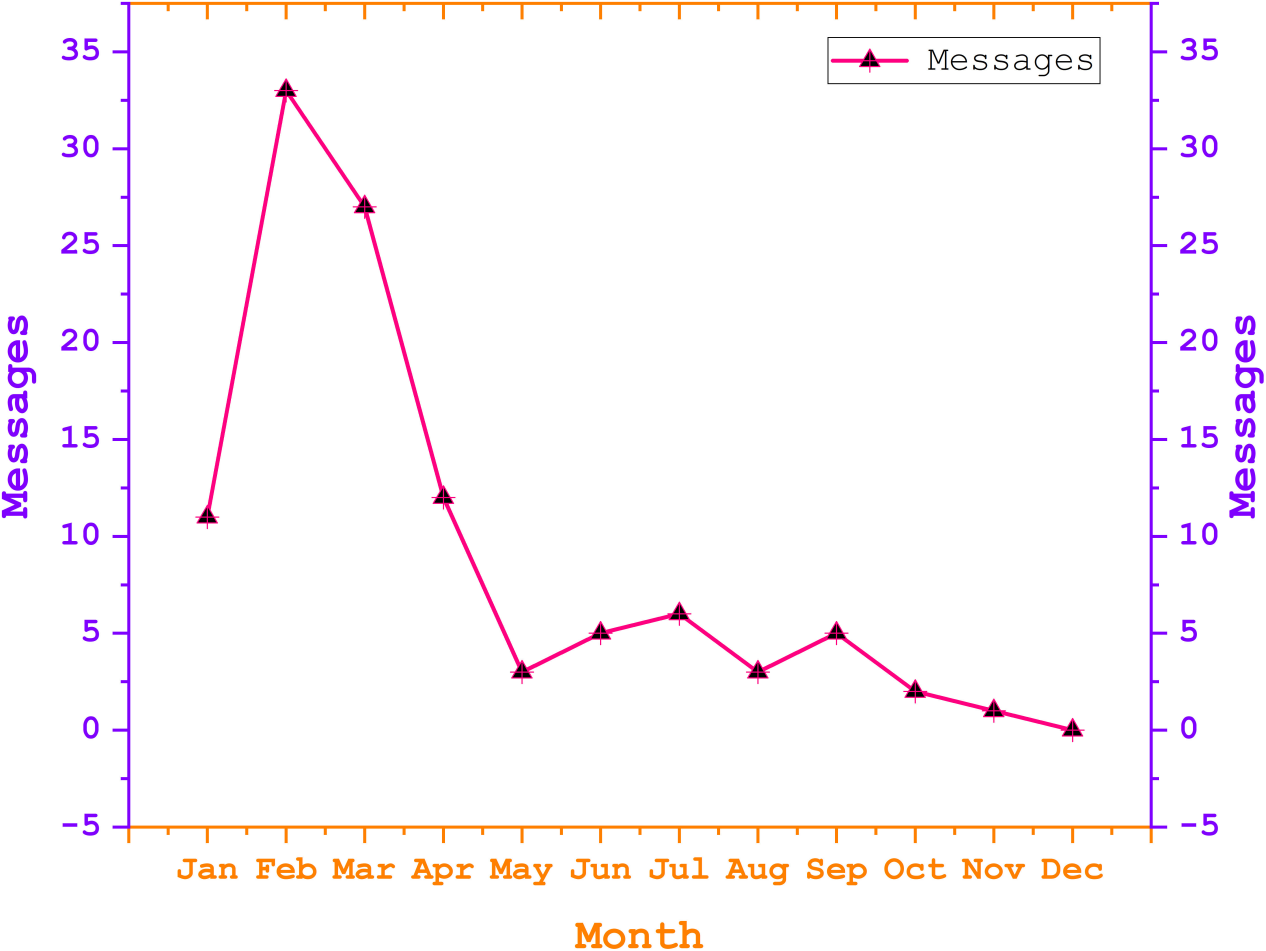
The trend chart for the quantity of SMS about the pandemic situation in Chongqing.

As can be seen in Figure 3, there were two phases in SMS outreach for pandemic prevention and control. One is from January to May 2020 and the other is from June to December 2020. We analyzed the interview results by stages.

From January to May 2020, the Chongqing municipal government sent a total of 86 short messages. We collated the time, department, content, and other data of these messages. Within 5 months, the number of push messages per month was 11, 33, 27, 12, and 3, respectively. The Chongqing Municipal Health Commission (CMHC) was the main push department, accounting for 72% of the total 62 SMS sent. The remaining 14 departments included, among others, the National Security Office (NSO), The Popularization Law Office (PLO), the Tourism Bureau, and the Transportation Bureau. The content of the short messages focuses on personal protection (daily mask protection, etc.) and policy requirements (such as standards for enterprises to resume work and production) during COVID-19.

Furthermore, by placing the above SMS in the context of COVID-19 in Chongqing, we found the following characteristics of SMS: (1) SMS focuses on the core content or key tasks of government policies and plays a preventive role in prompting. Among the 86 short messages sent, about 90% of their content is about reminding citizens of prevention and control behavior, which is consistent with the requirements for home quarantine and personal prevention and control advocated by national policy. Moreover, the content was pushed in the form of warm prompts. (2) The SMS was part of government policy and the rest of the government departments assisted. The department with the responsibility for the SMS is the CMHC. The CMHC led the other departments in the prevention and control of COVID-19. More than 50% of government departments were involved in message delivery in Chongqing. (3) The frequency of SMS is consistent with the different degrees of urgency required by government departments for events or policies. In other words, government departments will adjust the frequency of an SMS push according to the emergencies so that the SMS push is consistent with policy promotion and event governance progress. From May 2020, Chongqing City passed through the outbreak period and entered a relatively calm period.

As shown in Table 3, 11 messages were pushed for 10 days in January 2020, during which the first-level response to major public health events was launched in Chongqing. In February 2020, a total of 33 posts were pushed, with an average of 1.14 posts per day. During this period, Chongqing issued the policy of resuming production and work in batches and an orderly manner at the beginning of the month. At the same time, the prevention and control measures were implemented in the latter part of February in regions and counties that were categorized as low-risk, medium-risk, and high-risk regions. In March, Chongqing's status was adjusted as requiring a second-level response to a major public health event. The policy included guidance on the prevention and control of the pandemic when returning to work and the city. By May 2020, COVID-19 in Chongqing city and China was well under control. Therefore, the number and frequency of SMS were sharply reduced during that period. Public SMS were sent out during the two major festivals, Qingming Festival and Labor Day, as reminders of the requirements for pandemic prevention and control. From June to December 2020, Chongqing Municipal Government sent 22 short messages, a significant decrease in the number. The content was all around tips for warm weather, such as pay attention to daily protection, keep social distance, and so on. The main sender of SMS messages was the Chongqing Municipal Health Commission.

**Table 3:** Descriptive statistical analysis of SMS.

**Month**	**Quantity**	**Policy**	**Type**	**Main content**
Jan	11	Initiate level 1 response for major public health events	Suggestion	Chongqing Municipal Health Commission reminds you: For your health and the health of others, please consciously implement the requirements of the Level 1 response, protect yourself well and reduce your outings. One less party meal, and family and friendships will not be broken. (January 28, 2020)
				Chongqing Municipal Transportation Bureau Tips: For your health and the health of others, please minimize unnecessary traffic trips and wear a mask on public transport. (January 30, 2020)
Feb	33	Orderly resumption of production and work	Suggestion warning education	Chongqing Municipal Health Commission reminds you: According to the relevant provisions of the pandemic prevention and control, if you have come to Chongqing from other provinces, for your health, please be sure to stay at home or in centralized quarantine observation for 14 days. Do not go out at will, and if you have symptoms of discomfort, please seek medical attention or report to your community in a timely manner. Thank you for your support and cooperation! (February 10, 2020)
				Chongqing Municipal Emergency Management Bureau reminds you: that it is strictly forbidden for enterprises to resume production and work ahead of schedule in violation of regulations... Production and operation are not allowed without pandemic prevention and safety conditions. (February 11, 2020)
				Chongqing Municipal Health Commission suggests: Please obtain information on the pandemic and protection from authoritative sources. Do not believe misinformation that creates panic, and do not spread misinformation that creates panic. Take basic precautionary measures, maintain a normal lifestyle, eat a reasonable diet, exercise appropriately, and avoid negative emotions. (February 26, 2020)
Mar	27	Reduced to major public health event level 2 response	Suggestion warning education	Chongqing Municipal Health Commission tips: the recent increase in the number of people returning to Chongqing, the risk of bringing in and spreading the pandemic has increased. Continue to implement preventive and control measures, strengthen self-protection, strictly prevent the risk of importing the virus and local sporadic risk, and resolutely prevent a resurgence of the pandemic. (March 19, 2020)
				Warm tip: prevent and control the pandemic, keep in mind the rule of law, and act in accordance with the law when fighting the pandemic. The National Law Popularization Office. (March 10, 2020)
				Chongqing Municipal Health Commission reminds you: everyone is responsible for their own health and should consciously take personal responsibility for the prevention and control of the pandemic. (March 10, 2020)
Apr	12	The pandemic has been effectively prevented and controlled	Suggestion education	Chongqing Municipal Health Commission suggests: at the juncture when pandemic prevention and control continue to improve, it is important not to be careless, not to relax. Don't be, blindly optimistic or complacent. Maintain good hygiene habits, continue to strengthen self-protection, wear masks, wash hands frequently, ventilate more, disinfect often, don't share meals, don't crowd together. Work together to consolidate the hard-won prevention and control achievements. (April 12, 2020)
				The National Anti-Fraud Center reminds you: Recently, there are some unscrupulous persons pretending to be the Department of Disease Control and Prevention, Medical Insurance Bureau and other departments to sell masks and other pandemic prevention materials and implement telecommunication fraud. 96110 is the national anti-fraud warning number, please answer it promptly. (April 20, 2020)
May	3	Requirements for pandemic prevention and control during holidays	Suggestion education	Chongqing Municipal Culture and Tourism Bureau and Chongqing Municipal Health Commission remind you: In accordance with the requirements of pandemic prevention and control, and for the health of tourists, the city's tourist attractions are open strictly by reservation in limited time slots during the May Day holiday. There is no admission without reservation. Please make reservations in advance for time slots, receive health codes, stagger your travel, go cautiously to popular scenic spots such as Hongya Cave, Yangtze River Ropeway and Magzikou, wear masks when visiting, do not crow together, keep social distance and visit in an orderly and civilized manner. (May 20, 2020)
Jun–Dec	22	Tips for warm weather	Suggestion	Chongqing Municipal Health Commission suggests: as the weather starts to get hot, the use of air conditioning needs to be evaluated comprehensively. If office premises and public places are known to have cases of COVID-19, generally do not use central air conditioning. (June 27, 2020)
				Chongqing Municipal Health Commission suggests: if you have fever and respiratory symptoms, please go to a designated hospital promptly. The general public should continue to maintain good health habits such as wearing masks, washing hands regularly, having more ventilation, gathering less, not crowding together, and working together for prevention and control to prevent the spread of the pandemic. (August 4, 2020)

### Grounded theory analysis of SMS messages

After interview data collection was completed, the data analysis phase began. We adopted a procedural version of grounded theory, including open, axial, and selective coding for our data analysis.

#### Open coding

Open coding is a word-by-word analysis of interview sentences to refine meaningful concepts (Pieterse, 2011). First, we collected and organized the interview data, and compared and analyzed word-by-word and sentence-by-sentence to find key words and formulate labels. Then, by stripping the original materials layer-by-layer, we further abstracted them to form concepts with core contents. Finally, the concepts were classified around the core issues, and merged with each other to refine generalized categories. During this process, the researcher kept an open mind.

The coding was done without omitting any important information until saturation, at the same time looking for words in the interviewee's discourse to condense for analysis. Seventeen concept codes, and 13 core categories were obtained. After completing the interviews with ten people, no more new concepts emerged, and the theory was largely saturated. The open coding process was completed with the help of NVivo12 software and open coding local examples, as shown in Table 4.

**Table 4:** Examples of open coding analysis.

**Partial original statements**	**Concept codes**	**Categories**
Older people like us still mainly use SMS, and it is difficult for us to access information through multiple channels like younger people who are familiar with the internet. (Interviewee 1)	Sex, age, region, and occupation	Individual characteristics
The main concern of construction workers like us is when we can resume work. Short messages are still able to deliver news about the resumption of work in a relatively timely manner. (Interviewee 5)		
There are many news apps on mobile phones, Access to news on the COVID-19 pandemic is still relatively easy and comprehensive. (Interviewee 4)	Behavior on the internet	Information learning capability
I use an old-fashioned mobile phone (non-smart phone without internet access), I don't have internet access, and I don't know about the COVID-19 pandemic, so I can only listen to other people in the village or my children's news. (Interviewee 2)	Channels for information acquisition	
I think the COVID-19 pandemic is still dangerous and easily contagious, causing me to be emotionally tense as well. Most residents are still more concerned about their own safety. (Interviewee 7)	Emergency, serious, crisis, and dangerous	Stressful emotions
Later on, fewer SMS messages were sent and the news conveyed the message that the pandemic was effectively under control, so everyone was relaxed. (Interviewee 8)	Safe, relaxed	Sense of security
The main reason for this is that I don't know enough and I can't do anything about it. (Interviewee 6)	Disaster, panic, anxiety, and helplessness	Feelings of panic
Misinformation increases public panic about the COVID-19 pandemic, and such misinformation should be clarified and controlled in a timely manner. (Interviewee 10)		
SMS is essentially in line with the government's efforts to prevent and control the COVID-19 pandemic, and I can understand this type of service. (Interviewee 4)	Support, approval, and understanding	Sense of identity
Residents are able to support our work well in the prevention and control of the COVID-19 pandemic, regardless of the type of information service. (Interviewee 7)		
I have received short messages from the government, all of which are warm weather reminders. These messages can teach me how to do a good job of daily protection, and can be called up at any time to see, quite convenient. (Interviewee 6)	Warm weather tips	Suggestions by short messages
	Protection recommendations	
When we work in rural areas, we remind residents to pay attention to short messages on their mobile phones because they can receive short messages no matter what kind of mobile phone they have, even if it is an older model. (Interviewee 8)	Communication of information	Neutral short messages
For some time, misinformation about “salt can prevent COVID-19” spread, and our government sent short messages about “don't believe misinformation, don't spread misinformation, and pay attention to authoritative information”. Instead of making direct comments, the misinformation is clarified by the national authoritative media. (Interviewee 9)	Clarification of misinformation	Opinion-led short messages
Chongqing Municipal Bureau of Justice has sent a short message informing about an incident that jeopardized the prevention and control of the COVID-19 pandemic, conveying the need for legal responsibility for disrupting pandemic prevention and control. This short message had a great effect on creating a good environment for pandemic prevention and control. (Interviewee 10)	Notification of the incident	
I am more concerned about when I can resume work, short messages telling me what materials I need to prepare, how to pay attention to protection during the resumption of work, etc. (Interviewee 5)	Handling services	Educational short messages
Not long after the policy on resumption of work came out, a short message was sent about “whether to turn on the air conditioner in the office”, and we were equipped with knowledge and skills on how to ventilate the office if the air conditioner was turned on to prevent and control the pandemic. (Interviewee 10)	Protective skills	
At the beginning of the COVID-19 outbreak, the first channels to get information were TV news and short messages. Short messages were displayed directly on mobile phones without manipulation or internet access. (Interviewee 1)	Effective service, easy operation, and wide-ranging reception	SMS
I learned about the government's requirement for us to segregate at home mainly through official news and official Mini-Apps. (Interviewee 6)	Official, authoritative, relatively rich in content	Official media
I usually follow online news and social media more because it allows me to engage in interactive communication and it has many forms. Recently I have received short messages but have not read them in a timely and regular manner. (Interviewee 4)	Network news, social media, interactive communication	Social media

#### Axial coding

Axial coding is used to discover and establish associations between conceptual classes to describe the connections between parts of the material (Liu et al., 2020). By revisiting the research questions and repeatedly reading the source material, the main categories with convergent meaning are further integrated. After meticulously combing the 13 categories formed from the open coding for clustering and summarizing, the four main categories of emotional need, SMS function, individual difference, and media channel were formed. The main axis coding process and the meanings of the corresponding categories are shown in Table 5.

**Table 5:** Results of axial coding.

**Main category**	**Subcategory**	**Connotation of categories**
Individual differences	Individual characteristics	The individual's gender, age, region, occupation, etc.
	Information learning capability	Differences in individual knowledge and use of various media.
Emotional needs	Stressful emotions	Nervousness and helplessness.
	Sense of security	The public correctly understands the COVID-19 pandemic and maintains a stable and peaceful mindset.
	Feelings of panic	Crisis/emergency evaluation of the COVID-19 pandemic event as well as their own fear and panic psychology.
	Sense of identity	Recognition, support, affirmation and understanding of SMS.
Functions of SMS	Suggestive short messages	Warm tips and advice on individual behavior from short messages.
	Neutral short messages	No emotionally charged words in the content of SMS messages.
	Opinion-led short messages	For misinformation and violations during the COVID-19 pandemic, they are guided by warning and guidance SMS messages tweeted.
	Educational short messages	SMS teaches the public how to protect themselves effectively in their daily life and work
Media formats	SMS	SMS has a wide range of acceptance and is easy to operate and reliable.
	Official media	A portion of the public prefers official media, which is considered more authoritative and relatively rich in content.
	Social media	A segment of the public tends to choose social media for its efficiency and ease of communication.

#### Selective coding

**Figure 4:**
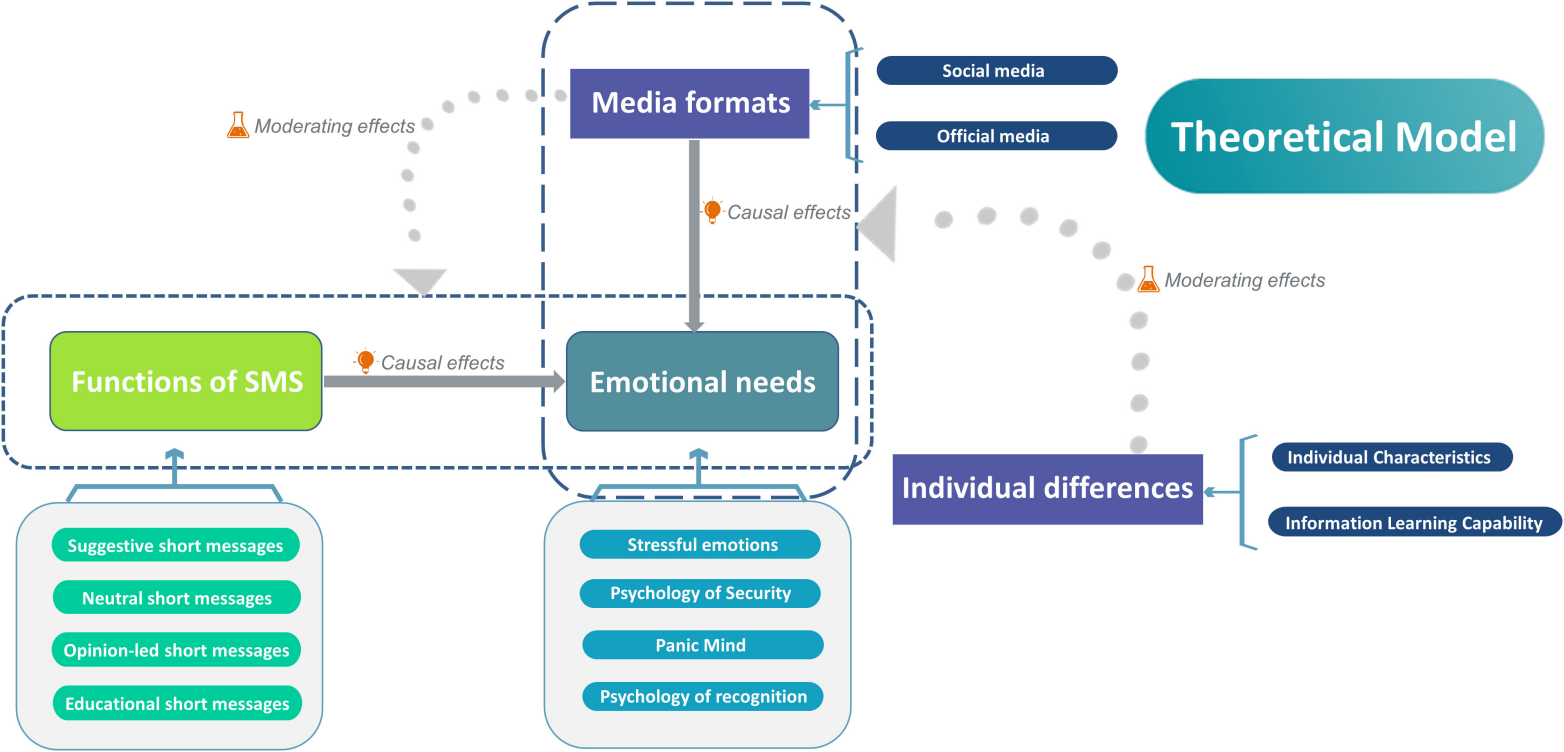
Theoretical model of SMS function to meet emotional needs.

Selective coding is used to systematically analyze the established conceptual categories and select a core category that is overarching and can link other categories into a whole to form a systematic theoretical framework. After analyzing all the discovered conceptual categories, we took “SMS function satisfies emotional needs” as the core category, studied the connections among the formed main categories from the perspective of logical relationships, clarified the storyline of information, described the main categories, sub-categories and their attributes and dimensions, and determined the selective coding path of the main categories in this study The relationships are shown in Table 6 and Figure 4.

**Table 6:** Results of selective coding.

**Typical relationship structure**	**Relationship type**	**Connotation of relational structure**
Functions of SMS → emotional needs	Causality	The different service functions of SMS meet and respond to the different emotional needs of the public.
Media formats → emotional needs	Causality	Differences in awareness and use of different media can satisfy different levels of emotional needs.
Media formats ↓ (Functions of SMS → emotional needs)	Moderating relationship	Differences in the public's awareness and choice of media affect the different demands for the functions of SMS, leading to different requirements for meeting emotional needs.
Individual differences ↓ (Media formats → emotional needs)	Moderating relationship	Individual differences lead to differences in the extent to which emotional needs are met when using the same media.

## Results

### SMS messaging supported the emotional needs of the public during COVID-19

From the above analysis of the data, it can be seen that the SMS in Chongqing during the outbreak of COVID-19 included content on government policy requirements for COVID-19 prevention and control, but they also provided support for the emotional needs of the public. As a traditional form of government communication media, SMS supported the public's emotions in three ways, which are described in the paragraphs below.

#### SMS suggestions attract the emotional attention of the public

The goal of government-led SMS is to promote government policies and governance of public events in educational and constructive ways that are closely connected to the daily life of the recipients, namely the public. The differences among the users of Internet-related platforms are a result of an information network infrastructure gap. Even though it is a subset of the Internet, SMS are no longer universally popular and their outlook is bleak. However, the new media forms are not accessible to everyone. Therefore, SMS delivery should be integrated into overall mobile communication technology to ensure that official policy information can be transmitted to all members of the public. While the integration of SMS with new media technology is in process, SMS can serve the preferences of different audiences by disseminating information through a short message format and suggested content. Traditional SMS does have the advantage of being able to deliver timely and effective information to the public. For example, in January 2020, most government text messages prompted users to “Pay attention to diet, go out and party less, and take personal protection.” The following excerpt is about one interviewee's assessment of the effectiveness of government governance by SMS during the COVID-19 pandemic.

In the early days of the outbreak, information about the outbreak had not yet reached a scientific conclusion. The government's work had two requirements. First, to make every member of the public as aware as possible and to participate in the prevention and control of the outbreak. Second, to tell them clearly and succinctly what to do. At this time, television and text messages could play this role very well (Interviewee 9).

#### SMS calm public nervousness with value-neutral text messages

SMS have the unique characteristics of neutrality and value. They do not evaluate public events or the administrative official notice but focus instead on how the information conveyed affects or interferes with social public life. Concerning official government propaganda media, SMS are ineffective administratively but incorporate the intrinsic value of official media. In particular, SMS are a traditional way of disseminating government information and are part of the government's administrative function. The content of SMS conveyed to the public is consistent with the government's views. However, this unity lacks administrative effectiveness and does not have the force of law. The value of the official message media channel is its neutrality. Specifically, the SMS form of media emphasizes the official requirements and value of the message but does not require a mandatory Yes or No response. Communication of information imparts value and is not a command. Short messages beginning with the words “warm tips” is an example of such communication. Excerpts from interview interviewees also provide feedback on government SMS—“In the early days of the outbreak, our community staff was highly stressed, not to mention other community residents,” (Interviewee 7) and….

When working in the countryside, we reminded residents to pay attention to short messages on mobile phones because no matter what type of mobile phone, they can receive text messages that persuade them to consciously isolate themselves at home. This dispels residents' resistance and calms down citizens' nervousness (Interviewee 8).

#### The use of public opinion to dispel public panic

Official SMS consists of official information from the government and is the government's response to social media. Official SMS has no decision-making significance and is another way to convey the government's policies or events. SMS also plays a key role in the government's information dissemination goal—to guide public opinion. Important decisions concerning major emergencies or the public's opinions on the government's social media are multifarious. The public's opinion of SMS guidance is apparent. The government's goals for SMS sent from its social media department are to instigate a public opinion response and to lead the mainstream with its social media propaganda. This is accomplished *via* the interpretation of major decisions or push to represent the government in decision-making. Therefore, based on the promotion of wide coverage and timely SMS dissemination, SMS have become the guiding standard of the government in social media propaganda and can deliver the government's authoritative information and attitudes to the grass-roots audience. For example, on February 14, 2020, a short message from the government warned that “if someone in the vicinity is diagnosed, take good precautions and do not panic.” On February 25, 2020, the government warned that a “rational response to the pandemic should not be excessive panic and anxiety.” On February 26, the government sent out an SMS that people in the “low-risk area could go out,” and pointed out rumors such as “blood pressure drugs increase the risk of disease,” and “onions can aid the adsorption of viruses to reduce risk” as false. The Chongqing municipal government's social platform released the messages “please get the pandemic information protection knowledge from authoritative sources and “don't believe misinformation, don't spread misinformation, either” on the same day. Interview excerpts from interviewees 1 and 5 also provide feedback on government SMS, as follows:

I think the most effective thing about a text message is that it's reliable. Especially when someone in the surrounding area is infected, you'll panic and not know what to do. You'll know what to do once you read the message. Although the community will help us to solve the problem, that is really after the fact. So, a first-time text message let me feel at ease (Interviewee 1).

Like us builders, we are relatively uneducated, and we are worried about our safety, but we can't tell when there are rumors, so we feel even worse. I thought it was really nice to clear up the rumor in a text message (Interviewee 5).

### Discarded SMS: a focus on “individuals with limited internet access” and the emotional needs for security during COVID-19

In June 2020, Chongqing's Covid-19 pandemic prevention and control outreach for COVID-19 entered a relatively calm period. From June 2020 to December 2020, the Chongqing municipal government sent out a total of 22 text messages, a significant decline in numbers from the preceding months. The SMS content included “warm tip” type messages on ways to pay attention to daily protection and maintain social distance. The main sender of the SMS push was the Chongqing Municipal Commission of Health.

Between June 2020 and December 2020, the role of SMS changed as the public's nervousness and panic over the outbreak eased after it was brought under control. At this juncture, authoritative information became abundantly available in the new media, and the SMS push was relegated to the discarded stage of its evolution as its effects on public emotion were replaced by new technology.

#### SMS devolves into an emotional support tool for “individuals with limited internet access”

The informational credibility of SMS affected users' behavior and attitudes (Ryan and Hayden, 2012). Credible and authentic informational messages to users can gain users' trust. This perception is the essence of SMS and can affect user's actions (McMahan et al., 1998; Taylor, 2009). In the interviews, we found that due to familiarity with the Internet, young people (regardless of gender and occupation), were to be able to derive the latest news about COVID-19. Concerning SMS, they said that they “did not carefully read;” “information is not much, feel useless,” or “it is more suitable for the traditional older people's use”(Interviewees 3 and 4), which are similar to findings of previous studies (Yousra et al., 2019). For older people, however, the opposite was true. Most older people said, “read (or heard) message.” There is a special mobile phone design for the elderly in China commonly known as the “Senior machine,” with large format fonts and voice broadcast SMS. Interviewee 1 said, “the feeling is very useful, to know how to prevent COVID-19.” Interviewee 2 said, “know how to arrange the pandemic prevention and control of the state.” Different benefits accrue from the wide coverage of SMS. In the research, we met an older person who returned from the remote mountain areas in Chongqing following his children. Interviewee 2 said “over there the signal is weak. Our village people have to go door to door disseminating prevention and control information. Many mobile phone short messages were sent and many were received, more than before. The state should pay attention to the pandemic because if people know the situation, they will also know how to cooperate.”

According to the 45th Statistical Report on Internet Development in China, in 2020, the number of Internet users in China will be 28.2% in rural areas; 16.9% of users will be people over 50 years old. This shows that rural areas and the older population are vulnerable targets for Internet development. In the face of public health crises, these groups can obtain authoritative and effective information through SMS. Compared with the information on the Internet, simple SMS content can meet COVID-19 prevention and control needs and provide access to information to a wide range of residents in a pandemic situation.

There are many kinds of SMS in China for the elderly and other unsure Internet users such as weather services, health services, and so on. But these services are based on the design and benefits of service providers. Services for public health emergencies such as COVID-19 are relatively weak with poor design for SMS. However, it is undeniable that during the COVID-19 prevention and control period, SMS played an important media role for elderly people, people with weak Internet access, and people living in remote rural areas and other areas with low Internet access.

#### The safe passage code of short messages replaced by the Yu Kang code (application small program) to meet emotional security needs

During COVID-19, the Ministry of Industry and Information Technology of the People's Republic of China held a press briefing with China's three telecom enterprises. They requested the telecom enterprises to provide customers with 14/15 days of visiting query services based on pandemic prevention and control requirements and telecom data analysis, with the user's authorization. Queries included the following: “Can you prove that you haven't been to a place with a severe outbreak in the last 14/15 days? Just send a text message to your carrier.” In China, as per the communication protocol, when each user handles the mobile phone SIM card and uses the communication services, the mobile phone terminal connects with the base station and verifies the user's identity (code and number information), and recognizes the communication routing and connection. In short, mobile phones have signals under normal conditions, the signal source is the base station, and each base station has a corresponding position. As the mobile phone moves, the base station of the signal source changes constantly and the base station records the mobile phone's access during the moving process, tracking the user's life trajectory. However, as per the needs of COVID-19, it is set at a 14/15-day time range for the query. The base station reflects the administrative area behind it but it can only reflect the general range. The precision range has not been captured so there will be no leakage of user privacy information.

Visiting query services can help relevant departments to improve the efficiency of itinerant inspection, conduct screenings on key groups, and carry out precise prevention and control to facilitate the resumption of work and production under the current situation. For example, China's mobile users first write CXMYD and send it to 10,086; they then list the provinces they have visited in the past 14/15 days by entering the four digits of their ID card based on the reply message to the appropriate municipal city.

This personal track serves as a passcode for users to return to factories and businesses during outbreaks. Interviewee 3, a migrant returning to work in Chongqing during the survey said “this method is easy to operate and can be operated without the Internet.” China has divided three risk areas into “high, medium, and low.” Users in high-risk areas are generally not encouraged to go out, while users in low-risk areas are discouraged from going to high-risk areas. Personal track services by SMS can visually display the areas that users have visited within 14/15 days and determine whether they can go back to work according to the level of risk. The information became an important safe passage code during COVID-19. But for the majority of the public, this security access code information has security problems. “It's a threat to your privacy to identify where you are by text message” (Interviewee 4).

After May 2020, Chongqing City released and popularized the Yu Kang Code small program. The program docks with data from the Chongqing Municipal Health Commission to protect privacy, authority, and security. The program contains the user's pandemic status (red QR code for infected, green QR code for security personnel, and yellow for close contacts). The function of the safe access code, the results of nucleic acid detection, and the vaccination were combined into a small program.

When asked to compare the safe access code with the Yu Kang code, interviewee 3 said that the “Yu Kang Code is additional authority and security,” interviewee 4 said “it can make people feel more at ease, and interviewee 6 said “I don't read text messages so much, it's convenient to travel with this code.” The Yu Kang Code is an effective application of big data technology. According to interviewee 9, “from the public's perspective, it is incomparable with other media such as short messages. It is easier to meet the public's needs for timely and authoritative information and information security.”

## Discussion and conclusions

The use of certain media in communication activities is influenced by inter-related media characteristics (Trevino et al., 1987). Specific communication conditions, perceptions of the purpose of the media, and the diversity of participants affect how the media is used. It defines the nature of the communication technology and media (Licoppe and Smoreda, 2005), and how well the different media cooperate rather than compete (Quan-Haase and Wellman, 2006). Hyo et al. (2007) studied the role of different media in Korea—a media group made up of SMS, E-mail, and Face to Face—and analyzed the distribution mode of different media. Hyo's study supports the need to research the role of SMS in the media structure and the government's pandemic management and intervention efforts concerning the public's informational and emotional needs.

### Comparison and links between SMS, official media and social media

From the perspective of the government's media structure, SMS, a traditional communication medium, together with official media and social media, has shaped the media communication environment and channels for public emotional support. The official media publicly and formally disseminates the government's decisions and/or opinions with administrative effectiveness. Social media is represented by various media platforms and by social or mobile clients in the current media integration space. The official media distribution channels and participants in the main body are relatively separate but their dissemination of information remains authoritative. The current channels are extensive distribution channels of various forms providing complex information for social public participation within the main body. Somewhere in between lies SMS.

Therefore, the interaction between SMS and the government needs to return to its media environment and the structure should be discussed. SMS forms a triangle of stable media structures with official media and social media. This structure is determined by the characteristics of the government's official information communications. Firstly, official government communications should guarantee the authority and authenticity of the information communicated, provide guidelines, and advocate mainstream values. Important decisions of significance to the government should provide an authoritative emotional support base for the public.

Secondly, official government information should be targeted at the public and should include broad coverage, receptivity of information, communication tools, content, and form.

Thirdly, as the media channel for disseminating information to the public, official government information needs to be differentiated from diversified and complicated social media content. Although the government should have limited intervention in social media, it should take into account the requirement, challenges, and benefits of social media so it can respond to the varying emotional needs of the public. Official media communicates authoritative information and values, whereas social media responds to information demand and provides social value feedback to the public. SMS is a conversation between the government and the public that provides neutral and objective suggestions and guidance. As an intermediary medium and source of emotional support, SMS disseminates official government information to the public far beyond social media. SMS is not only the authority for guiding public behavior in society, it can also specify the appropriate response to social media. The three—SMS, official media and social media—form a complementary triangle and an interactive communication structure.

In addition, during the pandemic prevention and control period, an interesting phenomenon was that government departments in some countries (including China, Japan and Italy) used loudspeakers to broadcast easy-to-understand prevention and control content in the streets and communities at grassroots level. The public felt the support of government and a collective cohesion through the use of vivacious voices. This inspired a positive mood in dealing with the pandemic and cooperating in prevention and control management. In particular, during the emergency phase of the containment and home quarantine policy in China, a multi-channel online/offline propaganda and communication model was established through radio, SMS announcements, telephone communication and WeChat to disseminate information and keep abreast of the physical and emotional needs of the public.

Government departments organized distribution of food and medicine, helped citizens with inconveniences and difficulties in an orderly manner, and opened a *green channel* (Medical, transportation and other departments set up easy procedures, safe and fast access) for patients with acute illnesses and pregnant women to seek medical treatment. This was also a reflection of the reality of the triangular media structure.

### Application of SMS messaging in public health crises in developing countries

From the perspective of the governance of public health emergencies and intervention to public sentiment, the interaction of SMS in the government's governance in developing countries focuses on the dynamic adjustment of information dissemination and the public's emotional needs.

This interaction also motivates the government to develop policy communication to address public health crises. The interactive role of SMS in the government's communication media structure focuses on the dynamic adjustment of media and information transmission. SMS is a combination of official, authoritative media, and social media, which requires it to adjust the media relationship between the two and maintain a benign media interaction. When the government faces a major decision or emergency, social media is superior to SMS in timeliness, but SMS is a complementary form of social media, which extends the information transmission range and coverage. It can especially serve older people who may have limited internet access.

SMS delivery of health information is beneficial for public health (Ybarra et al., 2014), and extends the scope and effectiveness of national governance. In community efforts to prevent dengue fever and rabies, the combination of SMS and regular information meetings can produce better results (Dammert et al., 2014; Wu, 2016; Ashmin et al., 2019). SMS is also effective as a control tool to reduce the malaria burden in children under 5 years (Aliyu et al., 2019). When social media is flooded with a variety of active voices and misleading content, SMS, as an extension of the authority of the official media, can respond with rules and guidance to ensure effective coordination between official media and social media.

SMS can help public health event governance and public emotional needs in developing countries, thanks to its ability to push our information and support (Lubis et al., 2019). Studies have found that SMS reduced negative emotions in the public (Bengtsson et al., 2015; Mao et al., 2016; Panigutti et al., 2017). SMS has gained new life in public health crisis management. It has brought action and a framework to governance based on its data superiority and capacity for providing emotional support. In the internet information age, this advantage is both irreplaceable and an innovation in governance. Mobile phone tracking technology is considered one of the most effective ways of identifying precise locations and information due to the widespread use of mobile phones by a large number of people (Lai et al., 2019). In the context of public health issues, mobile phone data can be used to predict a patient's movement trajectory and to identify the transmission source or area of the disease before implementing targeted control. Scholars have called SMS intervention “spatio-temporal personalization” (Yin et al., 2020).

### Research limitations and contributions

There are limitations in the data and analytical methods. Firstly, the government releases messages during important events or festivals in China. Due to inherent unpredictability, we were not able to obtain a whole year's data. We were also not able to obtain information on SMS sent by the governments of other provinces. Fortunately, the SMS messages we were able to obtain were consistent, that is they were supervised national government actions. Secondly, due to restrictions on access to SMS to protect personal privacy, we did not have access to information on the number of messages sent to every citizen. We can only conduct interviews on relevant short message topics in emergency governance research. In these interviews, we found that the effect of SMS was often ignored by interviewees. When we asked interviewees, they realized they had received short news messages and dozens of unread messages. We believe that these limitations don't affect the value of our research findings as we did not focus on the individual characteristics of users and usage habits, but only on the SMS. We found that the public's positive sentiments around the government's response to COVID-19 can be attributed to the positive impact of SMS.

Concerning the governance of COVID-19, the main conclusion of this study is that SMS had a positive impact on recipients. As a traditional media, SMS forms a triangle with the official media and social media, creating the government's communications structure. At the same time, SMS reshaped government emergency management, particularly in the initial stage of COVID-19. The emotional support offered by SMS to the public extended the scope of the government's emergency management. It also provided an effective channel for weaker uses of the internet to receive and perceive the efficiency and authenticity of government information. However, SMS now lags behind in form, content, and technology. We still need to understand the positive role this traditional media plays in meeting the public's emotional needs and the government's decision-making and governance of major public health crises. Changes in information technology and the current media environment can also enhance the significance of traditional media. An important topic for research into media development should be how to effectively identify the positive role of traditional media.

## Data availability statement

The original contributions presented in the study are included in the article/supplementary material, further inquiries can be directed to the corresponding authors.

## Ethics statement

Written informed consent was obtained from the individual(s) for the publication of any potentially identifiable images or data included in this article.

## Author contributions

MW and CW: conceptualization, data curation, supervision, and visualization. MW and XP: field research, validation, and writing-original draft. MW, CW, and XP: formal analysis. MW: writing-review and editing. All authors have read, agreed to the published version of the manuscript, and approved the final manuscript.

## Funding

This research was funded by Special Funds of the National Social Science Foundation of China (Grant Number 20VYJ031), Chongqing Technology and Business University High-Level Talent Research Start-up Project (Grant Number 2255003), the National Social Science Foundation for Young Scholars of China (Grant Number 22CGL034), and the Fundamental Research Funds for the Central Universities of China (Grant Number 2022CDJSKPY23).

## Conflict of interest

The authors declare that the research was conducted in the absence of any commercial or financial relationships that could be construed as a potential conflict of interest.

## Publisher's note

All claims expressed in this article are solely those of the authors and do not necessarily represent those of their affiliated organizations, or those of the publisher, the editors and the reviewers. Any product that may be evaluated in this article, or claim that may be made by its manufacturer, is not guaranteed or endorsed by the publisher.
